# SerpensGate-YOLOv8: an enhanced YOLOv8 model for accurate plant disease detection

**DOI:** 10.3389/fpls.2024.1514832

**Published:** 2025-01-20

**Authors:** Yongzheng Miao, Wei Meng, Xiaoyu Zhou

**Affiliations:** ^1^ School of Information Science and Technology, Beijing Forestry University, Beijing, China; ^2^ Engineering Research Center for Forestry-Oriented Intelligent Information Processing, Beijing, China

**Keywords:** plant disease detection, YOLOv8, complex environment, deep learning in agriculture, agricultural productivity

## Abstract

Plant disease detection remains a significant challenge, necessitating innovative approaches to enhance detection efficiency and accuracy. This study proposes an improved YOLOv8 model, SerpensGate-YOLOv8, specifically designed for plant disease detection tasks. Key enhancements include the incorporation of Dynamic Snake Convolution (DySnakeConv) into the C2F module, which improves the detection of intricate features in complex structures, and the integration of the SPPELAN module, combining Spatial Pyramid Pooling (SPP) and Efficient Local Aggregation Network (ELAN) for superior feature extraction and fusion. Additionally, an innovative Super Token Attention (STA) mechanism was introduced to strengthen global feature modeling during the early stages of the network. The model leverages the PlantDoc dataset, a highly generalizable dataset containing 2,598 images across 13 plant species and 27 classes (17 diseases and 10 healthy categories). With these improvements, the model achieved a Precision of 0.719. Compared to the original YOLOv8, the mean Average Precision (mAP@0.5) improved by 3.3%, demonstrating significant performance gains. The results indicate that SerpensGate-YOLOv8 is a reliable and efficient solution for plant disease detection in real-world agricultural environments.

## 1 Introduction

Facing significant challenges in plant disease detection and management within global agriculture, developing a highly accurate and efficient plant disease detection system is crucial for ensuring food safety and enhancing agricultural productivity. Traditional methods, which mainly rely on manual observation, are not only inefficient but also heavily dependent on the observer’s experience, making them unsuitable for large-scale agricultural production (Su et al., 2021; Vamsidhar et al., 2019).

Various published research papers have used image processing and deep learning-based techniques to classify infected plant leaves based on species and the diseases acquired. Aboneh et al. used pre-trained CNN models—InceptionV3, ResNet50, VGG16, and VGG19—to classify diseases in wheat plants. The dataset used is self-collected, and the images were captured in a natural environment (Wang et al., 2021). Due to this, improved accuracy was achieved under similar circumstances (Aboneh et al., 2021) Mohanty et al. used the GoogLeNet architecture and trained their model on Plant Village Dataset along with web-scraped images from Bing and Google. The vast variety of images present in the dataset leads to desirable results under laboratory conditions (Mohanty et al., 2016). Tete et al. have used various ANN-based classification techniques to compare results obtained based on recognition speed for a varying number of clusters present (Tete and Kamlu, 2017). Shrivastava and his team leveraged the AlexNet framework for feature extraction and then used a Support Vector Machine (SVM) approach to diagnose diseases in rice plants. The dataset they worked with consisted of images personally gathered from the Indira Gandhi Agricultural University in Raipur. Due to the dataset’s limited size, the model suffered from overfitting, a flaw apparent in their findings. This constraint is echoed in the study’s performance indicators (Shrivastava et al., 2019). On a different note, Mohanty and colleagues utilized the GoogLeNet framework for their research, training their model with the Plant Village Dataset enriched with images obtained through web scraping from Bing and Google. The extensive array of images in their collection led to favorable results in controlled experiments (Mohanty et al., 2016). Furthermore, Tete and associates investigated several classification techniques based on Artificial Neural Networks (ANN), examining how the number of clusters affects the speed of recognition. Their analysis provides valuable insights into the efficacy of various methods (Tete and Kamlu, 2017). Researchers have applied image processing and machine learning to identify and categorize plant diseases (Bao et al., 2022). Castelao Tetila et al. used six traditional machine learning approaches to detect infected soybean leaves captured by an Unmanned Aerial Vehicle (UAV) from various heights, validating the impact of color and texture features on the recognition rate (Castelão Tetila et al., 2017). Maniyath et al. suggested a classification architecture based on machine learning for detecting plant diseases (Ramesh et al., 2018).Ferentinos utilized simple leaf images of healthy and infected plants to construct convolutional neural network models for plant disease identification and diagnosis using deep learning (Ferentinos, 2018). Fuentes et al. employed “deep learning meta-architectures” to identify diseases and pests on tomato plants by utilizing a camera to capture images at varying resolutions, successfully detecting nine distinct types of tomato plant diseases and pests (Fuentes et al., 2017). Tiwari et al. introduced a dense convolutional neural network strategy for detecting and classifying plant diseases from leaf pictures acquired at different resolutions, addressing many inter-class and intra-class variances in images under complicated circumstances (Tiwari et al., 2021). Several other studies have utilized deep learning and image processing techniques to identify tea leaf diseases. Hossain et al. discovered an image processing method capable of analyzing 11 features of tea leaf diseases and used a Support Vector Machine (SVM) classifier to identify and classify the two most common tea leaf diseases, namely, brown blight disease and algal leaf disease (Hossain et al., 2018). Sun et al. improved the extraction of tea leaf disease saliency maps from complicated settings by combining Simple Linear Iterative Cluster (SLIC) and Support Vector Machine (SVM) (Sun et al., 2019). Hu et al. developed a model for analyzing the severity of tea leaf blight in natural scene photos, calculating the Initial Disease Severity (IDS) index by segmenting disease spot locations from tea leaf blight leaf images using the SVM classifier (Hu et al., 2021).

Moreover, notable architectures like AlexNet (Krizhevsky et al., 2012), VGGNet (Simonyan and Zisserman, 2014), GoogLeNet (Szegedy et al., 2015), InceptionV3 (Szegedy et al., 2016), ResNet (He et al., 2016), and DenseNet (Huang et al., 2017) have been used for plant disease identification.

In the context of object detection, the YOLO (You Only Look Once) algorithm, a widely used method in computer vision, processes images in real time via a single forward pass of a neural network, performing both object recognition and bounding box regression in one step. This efficiency allows it to process up to 60 frames per second. YOLO divides an image into a grid of cells and predicts bounding boxes along with class probabilities for each cell (Xue et al., 2023).To improve accuracy, YOLO uses anchor boxes of varying sizes and aspect ratios, which enhances its suitability for detecting multiple objects across various regions of an image (Jiang et al., 2022; Kirar, 2024).

Earlier versions of the YOLO family have been successfully applied in various domains, including for fruit identification in harvesting robots (Kuznetsova et al., 2020; Yan et al., 2021), vehicle and ship detection (Zhao et al., 2023; Song et al., 2023), and face detection (Ayo et al., 2022). Lidahua et al (Dahua et al., 2024) used an improved YOLOv7 for detecting apple surface defects. The enhanced model increased the detection mAP@0.5 by 2 percentage points compared to the original YOLOv7.

To address this challenge, this study utilized the PlantDoc dataset developed by the Indian Institute of Technology, comprising 13 plant species across 30 different states, representing both diseased and healthy conditions, which reflects the complexity of real-world agricultural environments (Singh et al., 2020). The YOLOv8 model’s C2F module was enhanced by integrating Dynamic Snake Convolution (DySnakeConv) (Qi et al., 2023; Xie et al., 2020), which significantly enhances the model’s capacity to detect fine details in elongated and twisted structures. Additionally, the traditional SPPF module was replaced with the SPPELAN technique (Wang et al., 2024), combining Spatial Pyramid Pooling (SPP) (He et al., 2015) and Efficient Local Aggregation Network (ELAN) (Wang et al., 2022), which significantly enhanced the model’s feature extraction and aggregation capabilities. This enhancement not only improved both the accuracy and robustness of the model but also lowered computational costs and accelerated inference speed. Moreover, the study introduced an innovative Super Token Attention (STA) (Huang et al., 2023) mechanism, which significantly enhanced the network’s ability to capture global features at early stages (Ding et al., 2022).

Ultimately, the improved YOLOv8 model was compared with other existing models to assess the effectiveness of the proposed improvements (Aliyu et al., 2020; Fu et al., 2021; Liu and Wang, 2020). The experimental results demonstrated that the enhanced YOLOv8 algorithm exhibited superior recognition performance in detecting plant diseases from images. Compared to the original model, the mean Average Precision (mAP@0.5) increased by 3.3%. These results demonstrate that the proposed plant disease image detection model exhibits excellent performance in various tests, offering an efficient solution for plant disease detection through the application and further refinement of these technologies.

## 2 Materials and methods

### 2.1 Data acquisition

The data used for modeling in this study were obtained from the PlantDoc dataset. Developed by researchers at the Indian Institute of Technology, the PlantDoc dataset represents a major advancement in the application of computer vision to agricultural challenges, particularly in the detection of plant diseases. This dataset fulfills the critical need for large-scale, in-field data, which is crucial for enhancing vision-based disease detection technologies.

The PlantDoc dataset comprises a comprehensive collection of 2,569 images, spanning 13 plant species and covering 30 distinct classes that reflect a spectrum of health conditions, from diseased to robust states. This meticulously curated dataset is the result of over 300 human-hours dedicated to annotating images sourced from a vast array of internet resources. It is designed to capture the complexity of real-world agricultural environments, highlighting diverse backgrounds and varying light conditions typical to farming regions, particularly in countries like India. The dataset has been tailored for practical application, ensuring compatibility with the lower-end mobile devices predominantly utilized by the local farming community.

### 2.2 Data preprocessing

In this study, the dataset was meticulously divided into training, validation, and test sets, with an 8:1:1 allocation. The training set contains 2,055 images, while the validation and test sets each contain 256 images. This distribution was designed to ensure a balanced representation of data across various stages of model development, while also meeting the requirements for model training and evaluation.

### 2.3 Improved YOLOv8 Model-SerpensGate YOLOv8

Obstructions from branches, leaves, and fruits often complicate the detection of plant diseases. Although existing deep-learning-based convolutional neural network models (Bi et al., 2022) achieve high accuracy, they remain constrained by high computational complexity and slow detection speeds. To address these challenges, this study introduces improvements to the YOLOv8 model (Redmon et al., 2016), with specific optimizations for plant disease detection in complex agricultural environments, ensuring accurate identification and monitoring.

**Figure 1:**
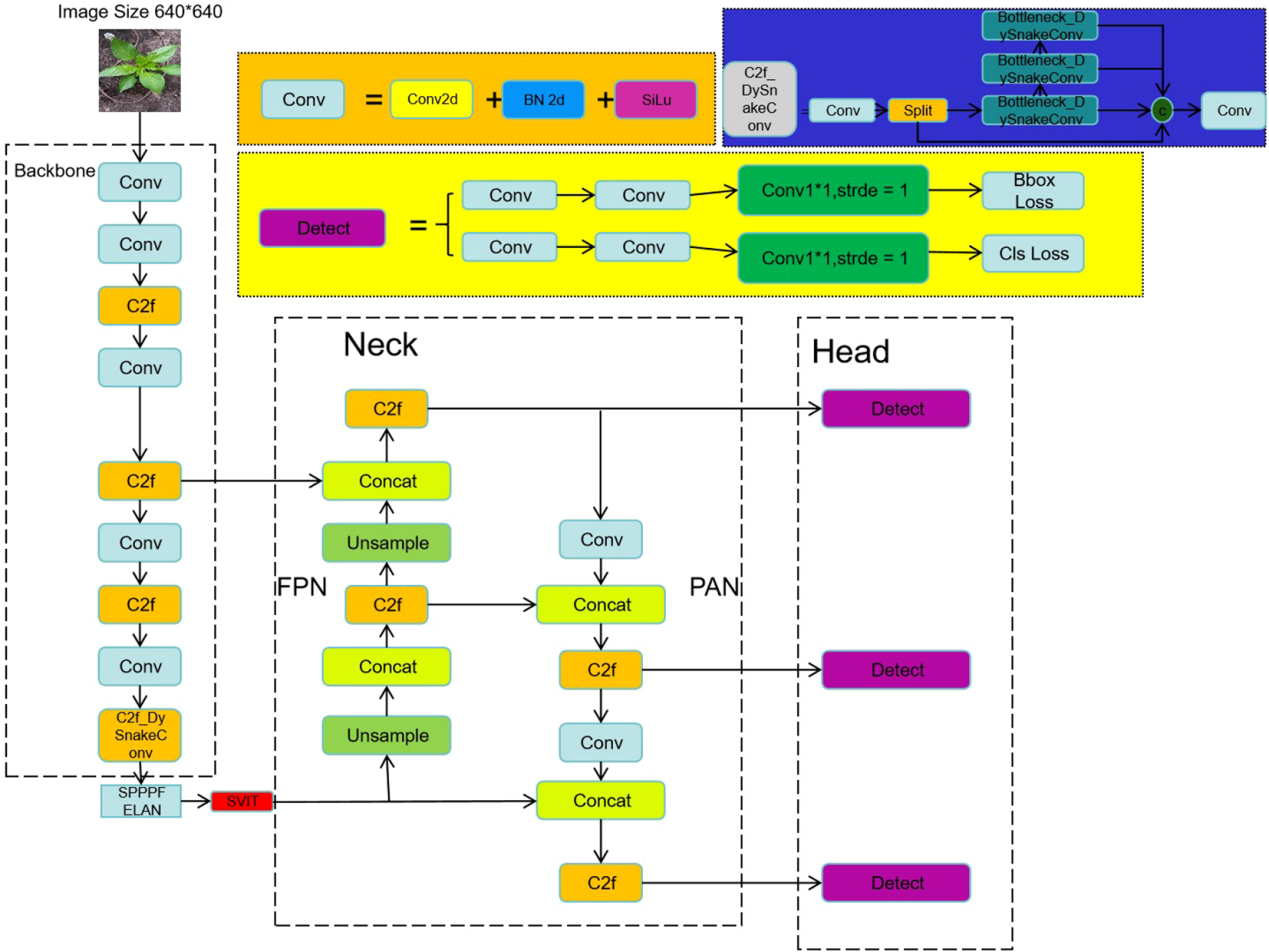
Model structure diagram.

The enhanced plant disease detection model based on YOLOv8 is illustrated in **Figure 1**. The model introduces modifications to the C2f module by adding more skip connections, removing convolutional operations in certain branches, and incorporating a split operation. These changes enrich feature information while reducing computational complexity, achieving a balance between efficiency and performance.

SerpensGate-YOLOv8 builds upon YOLOv8 by optimizing the ELAN (Efficient Layer Aggregation Networks), enabling richer feature extraction and precise detection of plant diseases in complex agricultural scenarios. The model’s input size is 640×640×3, and the input plant disease images undergo multiple convolutional operations and feature extraction. Through a combination of downsampling and feature concatenation, three basic feature maps are generated.

The top feature map is processed through Conv and C2f modules before being passed into deeper network layers to merge with features extracted from shallow layers. Meanwhile, the middle and lower feature maps are further refined using SPPELAN and DySnakeConv modules, ensuring effective feature extraction and integration across multiple network layers. After completing the top-down feature fusion, the network performs bottom-up feature fusion to further optimize feature representation.

The model produces three output feature maps of different sizes (20×20, 40×40, and 80×80). These multi-scale feature maps allow the detection of plant disease regions of varying sizes, effectively handling the multi-scale and complex morphological characteristics of plant diseases. This capability enhances the accuracy and robustness of plant disease detection. By predicting objects of different sizes using feature maps of corresponding scales, the model is well-equipped to process objects of varying sizes within the image.

In the SerpensGate-YOLOv8 object detection framework, the Backbone, Neck, and Head are the key components. Before entering the Backbone, the image data undergoes basic preprocessing steps, such as data augmentation. The Backbone is crucial for extracting features from target regions in input images. After passing through the Backbone, the data undergoes sequential processing through modules such as Conv, C2f-DySnakeConv, and SPPELAN. Subsequently, the STA attention mechanism further enhances these features, amplifying the weights of the target regions to extract more meaningful insights.

The Neck is responsible for feature fusion. Within the network architecture, three branches of different scales feed into the Neck, including the main branch enhanced by the STA mechanism. After feature fusion in the Neck, these three feature branches are passed to the Head for classification and detection of target features. The core function of the Neck is to integrate features of varying scales, improving the accuracy of target detection. Compared to the original YOLOv8, this model incorporates significant improvements in both the Neck and Backbone structures. Detailed modifications are annotated in the figure.

This study integrates innovative detection methods, including the Super Token Attention (STA), Dynamic Snake Convolution (DSConv), and Spatial Pyramid Pooling and Efficient Layer Aggregation Network (SPPELAN) modules. The STA mechanism decomposes visual information into “super tokens,”effectively reducing computational complexity while capturing global contextual information. This mechanism allows the model to focus on critical regions affected by plant diseases, enhancing robustness to complex backgrounds and improving the quality of feature representation. DSConv dynamically adjusts the shape and orientation of convolutional kernels, specializing in capturing irregular boundaries and intricate textures associated with plant diseases. This method demonstrates superior performance in detecting small or partially occluded diseased regions, significantly improving boundary detection accuracy and the model’s adaptability. SPPELAN employs multi-scale feature extraction and fusion mechanisms to accurately detect disease regions of various scales. By integrating local and global information through its multi-branch structure, SPPELAN reduces computational complexity via feature compression, enhancing both efficiency and accuracy.

The integration of these advanced modules not only strengthens the model’s ability to process complex image data but also ensures robust performance in diverse agricultural environments. These modules work synergistically to develop an automated system capable of significantly improving the precision of detecting, identifying, and classifying plant diseases. By enhancing diagnostic accuracy, this system saves valuable time for farmers, increases agricultural productivity, and ultimately improves their quality of life.

#### 2.3.1 Super token attention mechanism

The Super Token Attention (STA) mechanism (Huang et al., 2023) introduces a novel method to enhance the efficiency of global context modeling in Vision Transformers. This mechanism comprises three core processes: Super Token Sampling (STS), Multi-Head Self-Attention (MHSA), and Token Upsampling, each contributing to substantial reductions in computational complexity and improvements in performance.

In the STS process, we adapt the soft k-means based superpixel algorithm in SSN (Jampani et al., 2018) from the pixel space to the token space. Given the visual tokens $X\in {\mathbb{R}}^{N\times C}$ (where N=H×W is the token number), each token $X_{i}\in {\mathbb{R}}^{1\times C}$ is assumed to belong to one of m super tokens $S\in {\mathbb{R}}^{m\times C}$ , making it necessary to compute the X−S association map $Q\in {\mathbb{R}}^{N\times m}$ . First, we sample initial super tokens $S^{0}$ by averaging tokens in regular grid regions. If the grid size is h×w , then the number of super tokens is $m=\frac{H}{h}\times \frac{W}{w}$ . Then we run the sampling algorithm iteratively with the following two steps:

**Token & Super Token Association.** In SSN (Jampani et al., 2018), the pixel-superpixel association at iteration *t* is computed as


(1) $$Q_{ij}^{t}=e^{-\|X_{i}-S_{j}^{t-1}{\|}^{2}}.$$

Different from SSN (Jampani et al., 2018), we apply a more attention-like manner to compute the association map $Q^{t}$ , defined as


(2) $$Q^{t}=\text{Softmax}\;\left(\frac{XS^{t-1⊤}}{\sqrt{d}}\right),$$

where *d* is the channel number *C*.

**Super Token Update.** The super tokens are updated as the weighted sum of tokens, defined as


(3) $$S={\left({\hat{Q}}^{t}\right)}^{⊤}X,$$

where ${\hat{Q}}^{t}$ is the column-normalized $Q^{t}$ . The computational complexity of the above sampling algorithm is


**Figure 2:**
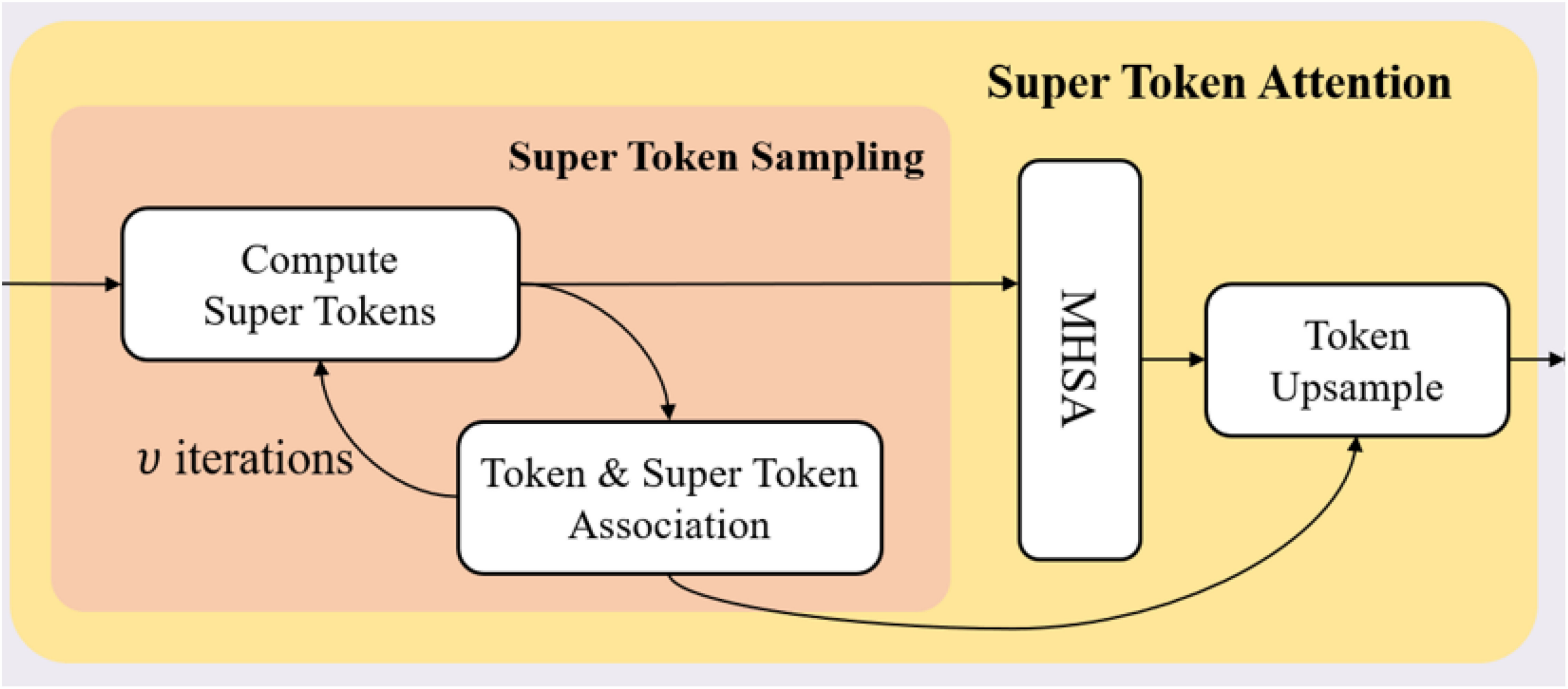
Super Token Attention.

(4) Ω(STS)=19NC,

where the complexities for obtaining initial super-tokens, computing sparse associations and updating super tokens are *NC*, 9*NC* and 9*NC*, respectively. We provide the details of the sparse computation of STA in **Figure 2**.

#### 2.3.2 Improvement of the backbone

In 2023, Yaolei Qi and colleagues introduced the DySnakeConv module (Qi et al., 2023), which demonstrated outstanding performance in recognizing tubular structures such as blood vessels and roads, tasks of comparable complexity to detecting plant diseases in agricultural applications. The DySnakeConv module specifically constrains the receptive field, ensuring the accuracy of convolutional deformations in alignment with the network’s overall loss metrics. Unrestricted convolutional flexibility could result in the loss of critical structural details in plant images. To address this issue, a continuity constraint was incorporated into the convolutional kernel design, allowing each position to reference the previous one while freely selecting the movement direction. This feature is essential for maintaining continuity in feature detection, particularly crucial for accurately identifying subtle plant diseases in diverse agricultural environments (Ma et al., 2021).

**Figure 3:**
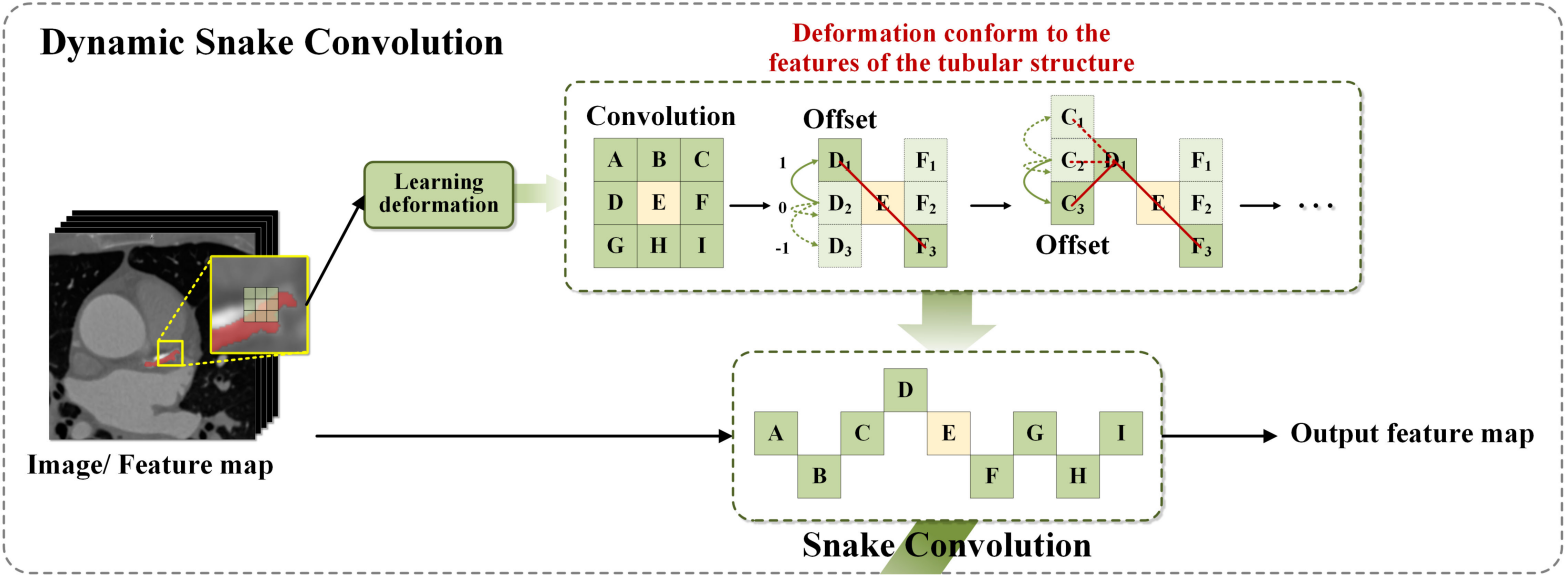
DSconv module structure diagram.

In the backbone architecture of the neural network, YOLOv8 employs the C2f module (Chen et al., 2023) as the primary component, specifically optimized to integrate low-level and high-level feature information for plant disease detection. The C2f module employs a densely residual structure to perform a series of convolutional operations, subsequently merging the information through splitting and splicing, effectively adjusting the channel count based on scaling coefficients to optimize computational efficiency and model capacity. This improvement significantly enhances the ability to extract features from elongated and complex lesions typical of plant diseases. DySnakeConv adapts to input feature maps, focusing on capturing complex and tortuous local features based on the morphology of the disease. By dynamically aligning with the actual morphology of lesions, DySnakeConv ensures more accurate and efficient detection of plant diseases, particularly those with irregular or changing forms.To further enhance the architecture, the Dynamic Snake Convolution (DySnakeConv) technique was integrated into the C2f module (Yu et al., 2017). In the original YOLOv8 feature fusion network, the C2f module had a fixed input size, supporting only input resolutions identical to those of the training images. Additionally, its reliance on fully connected layers for predictions introduced certain limitations in processing efficiency. To address these issues, the redesigned C2f_DySnakeConv module incorporates the DySnakeConv convolution, enabling adaptability to input images of varying sizes. This enhancement significantly improves the model’s capacity to handle diverse inputs while boosting the performance and speed of target detection.The C2f_DySnakeConv module introduces the following key improvements: 1.Replacement of traditional convolutional layers: The traditional convolutional layers were replaced with DySnakeConv layers, which dynamically adjust the kernel positions based on input image features. The module consists of two traditional convolutional (Conv) layers and two DySnakeConv layers. 2.Enhanced feature extraction: DySnakeConv is employed to capture diverse features of the target images, ensuring better adaptability to complex patterns. These enhancements greatly improve the ability to extract features from elongated and complex lesions commonly observed in plant diseases. DySnakeConv adapts to input feature maps, focusing on capturing intricate and tortuous local features based on the morphology of the disease. Layered computation across different feature levels not only optimizes resource utilization but also enables the effective detection of diverse targets. Using the Snake Model—a closed curve representing the lesion boundary—DySnakeConv dynamically adjusts through convolutional operations to closely align with the actual morphology of lesions. This capability is particularly advantageous for detecting plant diseases characterized by irregular, complex, or evolving patterns, thus enhancing the overall accuracy and effectiveness of the network in plant disease imaging. For a detailed description of the DySnakeConv module’s design and functionality, refer to the **Figure 3**.

#### 2.3.3 Improvement of the neck

The SPPELAN is an innovative network structure that effectively combines the advantages of Spatial Pyramid Pooling (SPP) and Efficient Layer Aggregation Network (ELAN). SPP, as a spatial pyramid pooling method, captures information at different scales, significantly enhancing the robustness of the model. On the other hand, ELAN improves the model’s representational capacity by efficiently integrating features from different layers of a deep neural network.

More specifically, SPP can handle inputs of varying sizes through its multi-level pooling, which incorporates pooling operations of various sizes (such as 1x1, 2x2, 4x4). This hierarchical pooling structure allows it to capture image features from multiple scales and ensures the output of a fixed-size feature vector. This is achieved by performing pooling over different regions at each level, then flattening and concatenating these features.

On the other hand, ELAN improves the efficiency of feature utilization by establishing direct connections between different network layers. These cross-layer connections enable features from lower layers to be transmitted directly to higher layers, enhancing the flow and reuse of information. During the feature fusion process, ELAN may also employ attention mechanisms or other strategies to dynamically adjust the weights of features from different layers to optimize the effectiveness of feature fusion.

**Figure 4:**
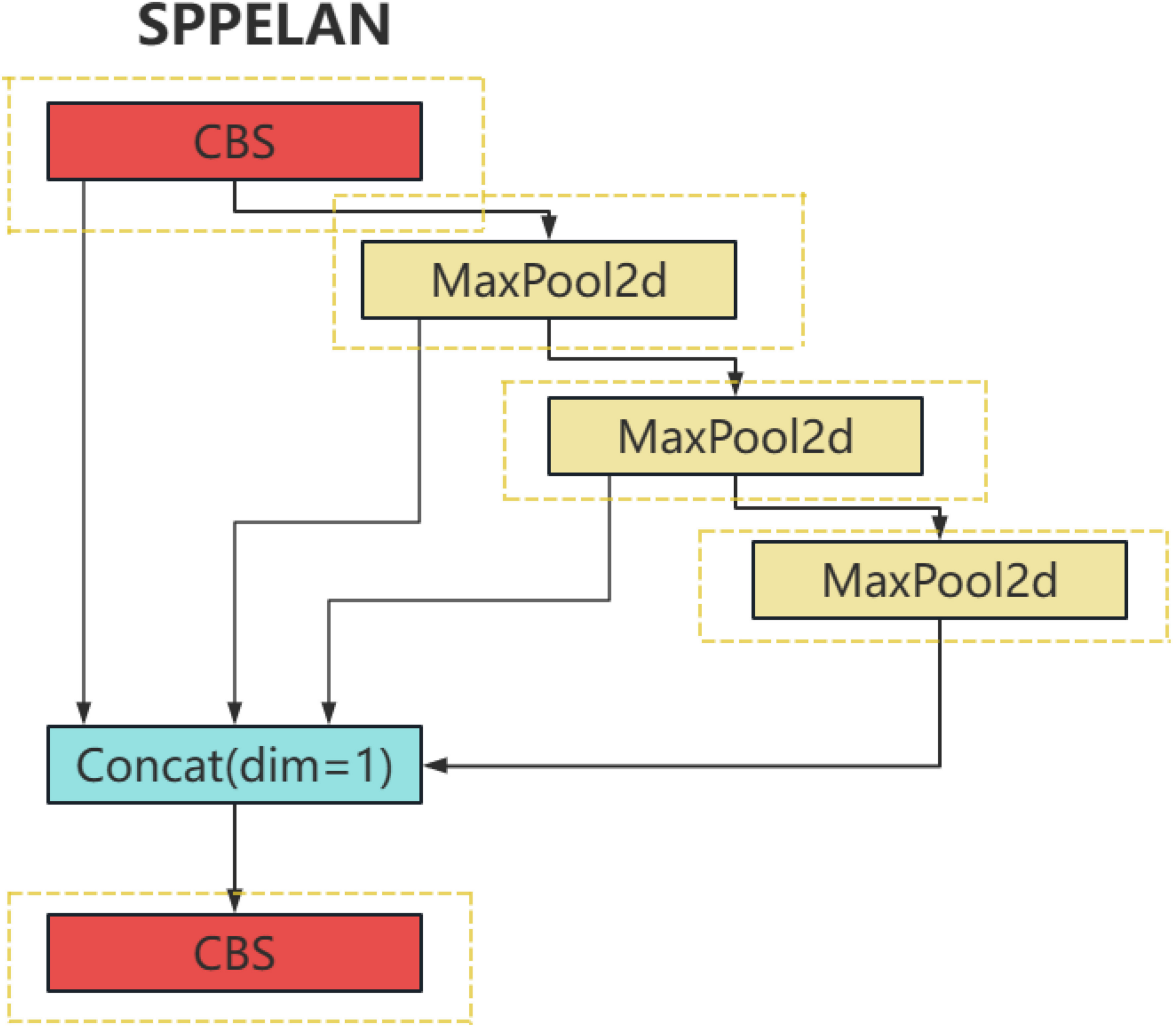
DSconv module structure diagram.

By integrating SPP and ELAN, the SPPELAN architecture captures more contextual information at various scales, thereby improving the accuracy of plant disease detection. This design not only enhances the model’s ability to handle agricultural images of varying sizes and resolutions but also optimizes the flow and integration of information within the network, making it particularly effective in identifying subtle and complex disease patterns in plants. The hierarchical pooling of SPP ensures the detection of disease features at different scales, such as small lesions or widespread discoloration, while the cross-layer connections in ELAN facilitate the seamless fusion of high-level semantic features and low-level spatial details. These capabilities significantly boost the model’s performance in the challenging task of plant disease identification. For a visual representation and clearer understanding of the SPPELAN module structure, refer to **Figure 4**.

#### 2.3.4 Model input

In the development of the plant disease detection model, the input image size was standardized to 640×640 pixels to accommodate SerpensGate-YOLOv8. This size was chosen to balance the demands for real-time processing and model accuracy, ensuring that the model not only preserves crucial image information but is also suitable for deployment on edge devices. Regarding data augmentation strategies, the approach used in SerpensGate-YOLOv8 is closely aligned with that of YOLOv5. However, a significant modification was made in the last 10 epochs of training for SerpensGate-YOLOv8: the Mosaic augmentation was discontinued (Castelão Tetila et al., 2017)

#### 2.3.5 Network Structure and Parameters

**Table 1** provides detailed insights into the network architecture and specific parameters of SerpensGate YOLOv8, enabling readers to gain a thorough understanding of its structural intricacies and design nuances.

**Table 1:** Network structure and parameters of SerpensGate-YOLOv8.

Layers	From	N	Params	Module	Arguments
0	-1	1	464	Conv	[3, 16, 3, 2]
1	-1	1	4672	Conv	[16, 32, 3, 2]
2	-1	1	18888	C2f	[32, 32, True]
3	-1	1	18560	Conv	[32, 64, 3, 2]
4	-1	2	134800	C2f	[64, 64, True]
5	-1	1	73984	Conv	[64, 128, 3, 2]
6	-1	2	507024	C2f	[128, 128, True]
7	-1	1	295424	Conv	[128, 256, 3, 2]
8	-1	1	982088	C2f_DySnakeConv	[256, 256, True]
9	-1	1	1313280	SPPELAN	[256, 256, 1024]
10	-1	1	262562	StokenAttention	[256]
11	-1	1	0	Upsample	[None, 2, ‘nearest’]
12	[-1, 6]	1	0	Concat	[1]
13	-1	1	148224	C2f	[384, 128, 1]
14	-1	1	0	Upsample	[None, 2, ‘nearest’]
15	[-1, 4]	1	0	Concat	[1]
16	-1	1	37248	C2f	[192, 64, 1]
17	-1	1	36992	Conv	[64, 64, 3, 2]
18	[-1, 12]	1	0	Concat	[1]
19	-1	1	156416	C2f	[448, 128, 1]
20	-1	1	147712	Conv	[128, 128, 3, 2]
21	[-1, 9]	1	0	Concat	[1]
22	-1	1	493056	C2f	[384, 256, 1]
23	[16, 19, 22]	1	757162	Detect	[30, [64, 128, 256]]

Summary: 329 layers, 4982500 parameters, 4982322 gradients, 9.7 GFLOPs.

### 2.4 Model train and evaluation

During the dataset annotation process, particular emphasis was placed on the accuracy and consistency of labels to ensure the development of robust models for plant disease recognition and severity assessment. These nuanced variations in the dataset annotation underscore the customized approach taken for this specific task.

The plant disease detection model constructed in this study falls under the category of target detection. After completing the model’s construction, key performance indicators such as Precision, Recall, F1-score, and mAP50 are used to assess its performance. Notably, AP refers to Average Precision. The specific formulas used to calculate these performance metrics are presented below. Here, TP, FP, FN, and TN correspond to true positive, false positive, false negative, and true negative, respectively. The variable C denotes the total number of categories, while APi represents the Average Precision value for the i-th category.


(5) $$\text{Precision}=\frac{TP}{TP+FP}\times 100\%$$

(6) $$\text{Recall}=\frac{TP}{TP+FN}$$

(7) $$\text{F}1-\text{score}=2\times \frac{\text{Recall}\times \text{Precision}}{\text{Recall}+\text{Precision}}$$

(8) $$\text{mAP}=\frac{\sum_{i=1}^{C}\;AP_{i}}{C}$$

where *TP*, *FP*, *FN*, and *TN* represent true positive, false positive, false negative, and true negative, respectively. *C* denotes the total number of categories, and $AP_{i}$ represents the AP value of the i-th category.

## 3 Experimental results

### 3.1 Experimental environment

To validate the effectiveness of the proposed methodology, an experimental setup was established using Ubuntu 18.04 as the operating system and Pytorch 2.2.1+cu121 as the deep learning framework. YOLOv8n was selected as the baseline network model. Detailed specifications of the experimental environment are presented in **Table 2**.

**Table 2:** Hardware and software configuration for training.

Environmental Parameter	Specification
Operating System	Ubuntu 18.04
Deep Learning Framework	PyTorch
Programming Language	Python 3.8
CPU	Intel(R) Core(TM) i9-12,900K, 16 cores, 24 threads, 3.19 GHz
GPU	GeForce RTX 3090Ti (24GB)
System Memory	32GB

To ensure fairness and consistency across model evaluations, all ablation experiments and comparative model training processes were conducted without the use of pre-training weights. Consistent hyperparameters were applied throughout the training across all experiments, ensuring uniform conditions for comparison. **Table 3** details the exact hyperparameters employed during these processes.

**Table 3:** Training parameter setting table.

Parameters	Setup
Epochs	500
Batch Size	8
Optimizer	SGD
NMS IoU	0.7
Initial Learning Rate Final Learning Rate Momentum	1×10^−2^ 1×10^−4^ 0.937
Weight-Decay Image Scale	5×10^−4^ 0.5
Image Flip Left-Right	0.5
Mosaic	1.0
Image Translation	0.1
*α* (Wise-IoU)	1.9
*β* (Wise-IoU)	3
Close Mosaic	Last 10 epochs

### 3.2 Ablation experiment

To thoroughly evaluate the enhancement in model performance, we defined four different configurations: the benchmark Model A, enhanced Model A + B (STA), enhanced Model A + B + C (neck, STA), and enhanced Model A + B + C + D (neck, STA, DSconv). These improvements and their impact were quantitatively analyzed based on metrics such as precision rate (P), recall rate (R), average precision (AP), mean value of average precision (mAP), number of parameters, and model size. The experimental results highlight the performance of these models on the test set, with detailed information presented in **Tables 2** and **3**.

### 3.3 Comparison of modeling results of classical object detection methods

Firstly, a comparative analysis of various classical methods based on the plant disease dataset was performed to determine the optimal modeling approach. The detailed modeling results are presented in **Table 4**. **Table 4** presents the results of our model in comparison with previous work on the PlantDoc dataset. Shill et al (Shill and Rahman, 2021). achieved mean Average Precision (mAP) scores of 53.08% and 55.45% using YOLOv3 and YOLOv4, respectively. Li et al. tested YOLOv5s (Li et al., 2022), nanodet-plus, and their own improved YOLOv5 model, achieving mAP scores of 53.5%, 55.3%, and 58.2% on the PlantDoc dataset, respectively.

**Table 4:** Comparison results on PlantDoc.

Methods	mAP(IoU=50%)	Recall(%)	Parameters(M)
M YOLOv4 (Shill and Rahman, 2021)	0.5545	56.0	–
YOLOv3	0.5308	57	15.3
YOLO v4	0.5545	61	62
YOLOv5s (Jocher, 2020)	0.5350	53.0	7.1
Improved YOLOv5	0.5820	55.0	8.4
nanodet-plus (RangiLyu, 2021)	0.5530	54.2	8.4
yolov8	0.6160	64.2	11.2

The relatively low precision, recall, and mean Average Precision (mAP) observed are attributed to the characteristics of the dataset. The images within the dataset feature objects set against natural backgrounds, which hampers the model’s ability to generalize across varying backdrops. Furthermore, the presence of multiple objects within these images introduces further challenges for object detection. The dataset utilizes images with dimensions of 416x416 pixels, making it particularly difficult to detect smaller objects effectively.

As shown in **Table 4**, a qualitative comparison indicates that YOLOv8 has significant advantages in constructing plant disease detection models. While its performance surpasses other methods, its parameter size has only slightly increased compared to YOLOv5 (Zhang et al., 2022). Therefore, this study will focus on improving subsequent models based on YOLOv8.

### 3.4 Modeling results of the plant disease detection model based on the improved YOLOv8

In Section 3.2, we explore the plant disease detection model based on the PlantDoc dataset. This section demonstrates the effectiveness of SerpensGate-YOLOv8 in identifying and classifying plant diseases. This section details the plant disease detection model built using SerpensGate-YOLOv8, with specific results shown in **Table 5**.

**Table 5:** Results of plant disease detection model construction.

Metric	Value
F1 Score	0.6120
Precision	0.7200
Recall	0.6620
mAP50	0.6490

After training our model, we used 239 images from each category in the dataset for testing.Below are the top ten results with the highest mean Average Precision (mAP) across all categories. **Table 6** details the specific performance of our model on both the training and testing sets.

**Table 6:** Performance comparison of training and testing sets (Top 10).

Category	Labels	Training Set						Testing Set	
		P	R	mAP@0.5	mAP@0.95	P	R	mAP@0.5	mAP@0.95
Strawberry Leaf	30	0.918	1.000	0.995	0.833	0.916	1.000	0.995	0.832
Grape Leaf	8	0.835	0.875	0.955	0.819	0.821	0.875	0.955	0.804
Raspberry Leaf	17	0.657	1.000	0.964	0.827	0.656	1.000	0.957	0.831
Corn Rust Leaf	10	0.816	1.000	0.931	0.740	0.762	1.000	0.931	0.740
Peach Leaf	10	0.763	0.900	0.917	0.706	0.631	0.900	0.918	0.694
Apple Rust Leaf	11	0.697	0.627	0.774	0.510	0.642	0.636	0.767	0.508
Corn Leaf Blight	12	0.712	0.823	0.777	0.674	0.662	0.833	0.767	0.665
Apple Leaf	10	0.455	0.700	0.733	0.566	0.442	0.700	0.722	0.556
Soybean Leaf	20	0.717	0.650	0.779	0.697	0.688	0.650	0.776	0.694
Blueberry Leaf	22	0.635	0.727	0.696	0.519	0.604	0.727	0.673	0.503

Due to the complexity of the plant disease detection data collected in this study, the primary issue faced during model training is overfitting. Therefore, we have plotted the relevant curves during the training and validation process and provided specific results.

**Figure 5:**
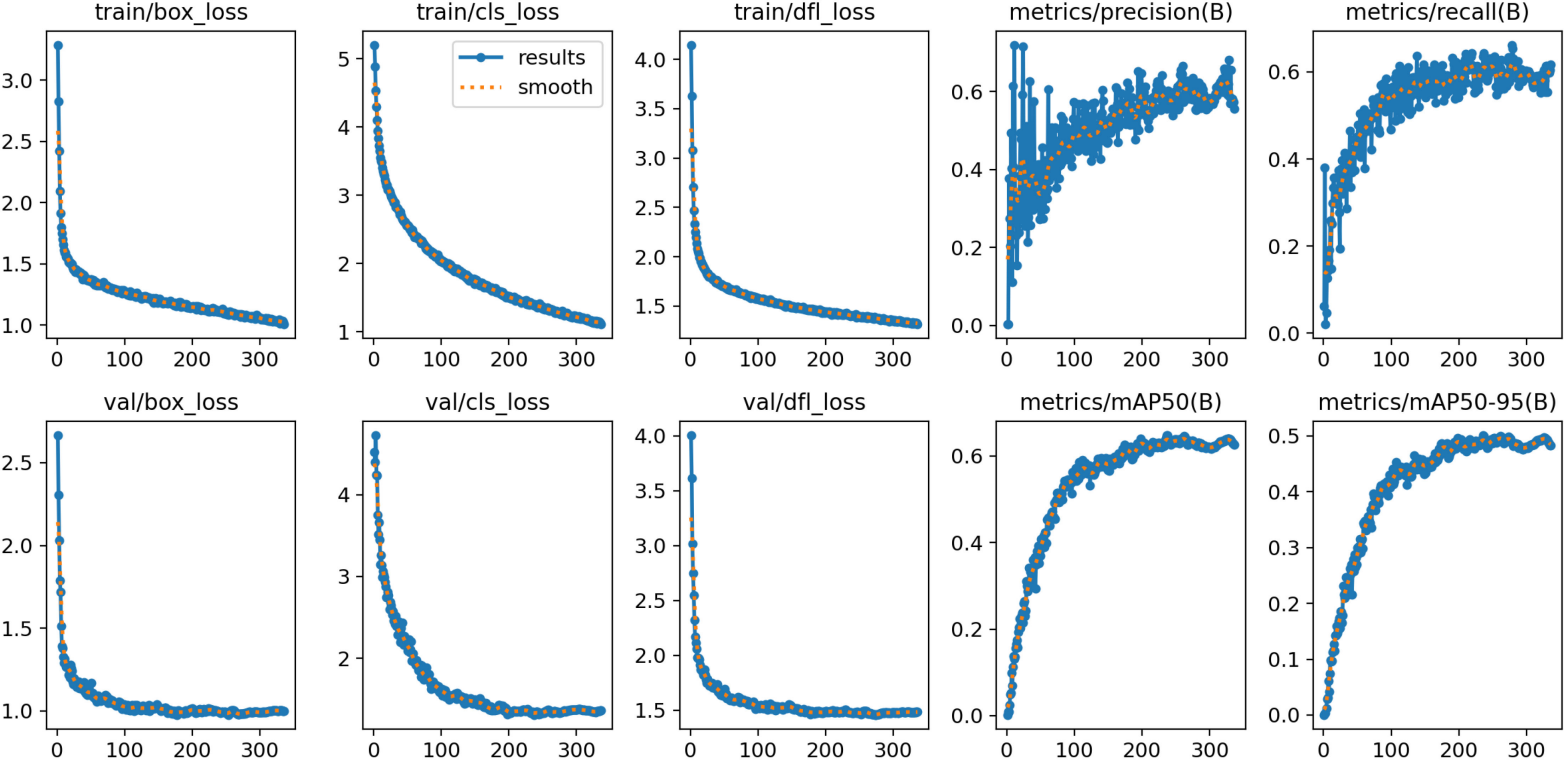
Training and validation process curves of the plant disease detection model.

As shown in **Figure 5**, during the training and validation phases, the loss curve exhibits an initial rapid decline followed by a gradual stabilization, while the performance metrics such as Precision, Recall, and mAP demonstrate a trend of rapid initial improvement and subsequent stabilization. This indicates that in the process of developing a plant disease detection model using SerpensGate-YOLOv8, there is no overfitting, and the model exhibits satisfactory convergence.

**Figure 6:**
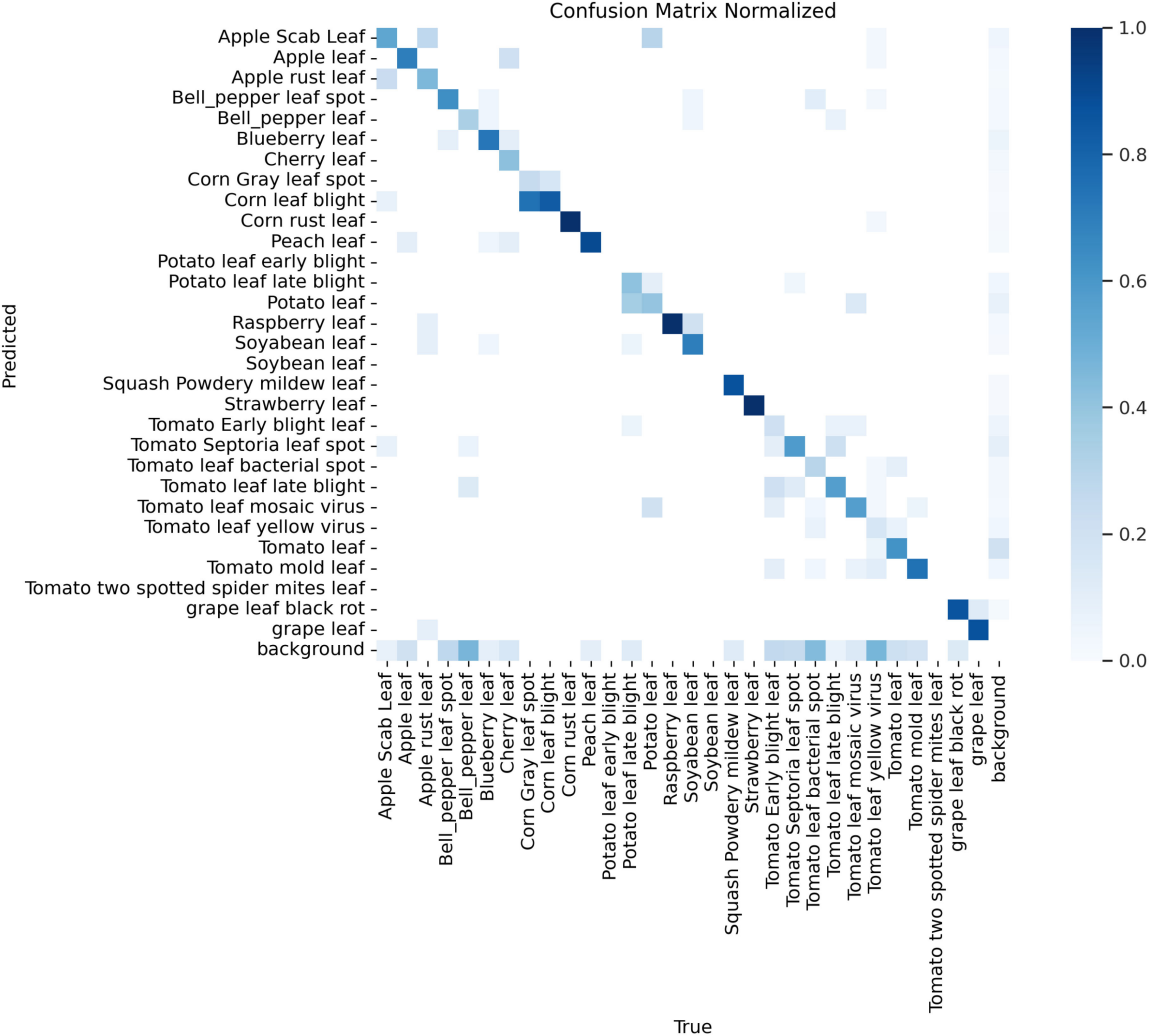
Confusion matrix of the plant disease detection model.

To visually demonstrate the performance of the SerpensGate-YOLOv8 plant disease detection model in target category recognition, this study has created a confusion matrix for the model, which is displayed in **Figure 6**.

According to the confusion matrix shown in **Figure 6**, the model is employed to differentiate between healthy plants and specific plant diseases. The matrix shows that the model exhibits high accuracy in identifying healthy plants, with 8 out of the top 10 highest recognition rates being for healthy plants. Additionally, the model also shows high accuracy in detecting certain diseases, such as corn rust leaf and apple rust leaf, as indicated by the darker colors on the diagonal, which indicates a high recognition rate in these categories.

**Figure 7:**
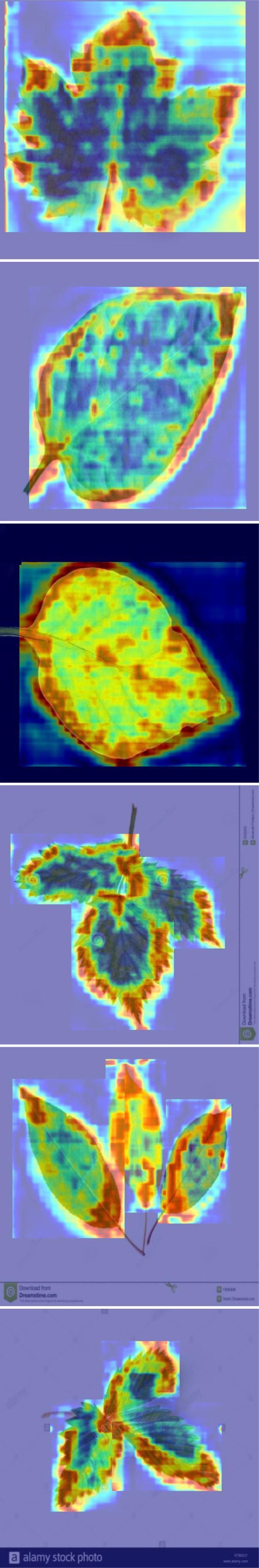
Grad-CAM visualization.

**Figure 8:**
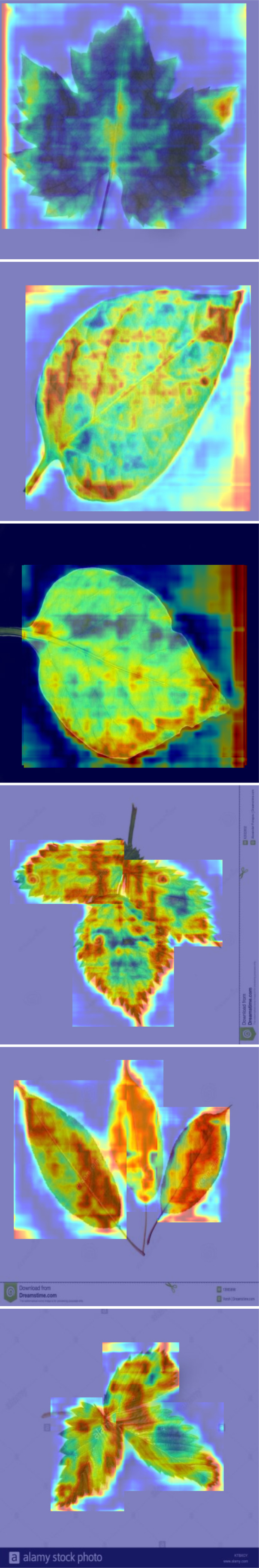
Grad-CAM visualization.

However, some categories remain challenging, such as tomato yellow virus and tomato Septoria leaf spot. Due to the similarities in color and texture features of these diseases, the model tends to misclassify these diseases. For instance, the similarity in growth stages and symptom presentations between tomato Septoria leaf spot and tomato late blight, combined with external environmental factors such as changes in lighting conditions, can increase the visual similarity between these two diseases, thereby impacting the model’s classification accuracy. These issues are reflected in the confusion matrix by the increased off-diagonal elements, indicating discrepancies between predictions and actual classifications. Therefore, further optimization of feature extraction and classification algorithms during model training is required to reduce the misclassification rate. After thoroughly discussing the performance shown in the confusion matrix on the PlantDoc dataset, we used Gradient-weighted Class Activation Mapping (Grad-CAM) to visually inspect the attention regions of the two models. **Figures 7**, **8** illustrates the category confusion between the two models.

In this study, we conducted experiments using both pre- and post-improvement models for plant disease detection. We found that these models exhibit varying performance in handling large categories, with particular challenges in distinguishing between background and foreground. By classifying and identifying different plant leaves such as apple scab leaves, apple rust leaves, and blueberry leaves, we observed significant differences in detection effectiveness. For example, the detection of apple scab leaves was satisfactory, whereas the detection of tomato late blight leaves was less effective. To explore the reasons for these performance differences, we introduced the Grad-CAM technique in the experiments. This technique allows us to interpret model behavior by visualizing the regions of interest where the model’s attention shifts. Grad-CAM calculates weights using the gradients of class confidence scores computed during backpropagation. These weights encode detailed class-specific information and are crucial for understanding the model’s decision-making process. Especially when detecting different types of plant leaves, such as apple rust leaves and tomato leaves, Grad-CAM reveals the precise regions where the model focuses its attention.

When using the YOLOv8 model, the performance was poor, with restricted focus on similar targets, particularly in cases of occlusion and small targets. In contrast, the improved model developed in this study demonstrated the best Grad-CAM visualization effects. We observed that some models concentrated on the edges, while others focused on the whole area. The deep red areas were distinctly visible, corresponding precisely to the target category. When detecting apple scab leaves and tomato late blight leaves, this model was able to fully cover the target areas in the image, showing high sensitivity to small and partially occluded targets. The experimental results indicate that the larger the plant disease target, the more complete the features learned by the network, and the better the model’s performance. This model has a significant advantage in detection accuracy across various categories. Additionally, the model’s processing speed analysis showed an average preprocessing time of 0.4ms per image and an inference time of 5.9ms, demonstrating that the model’s response speed is acceptable for practical applications.

**Figure 9:**
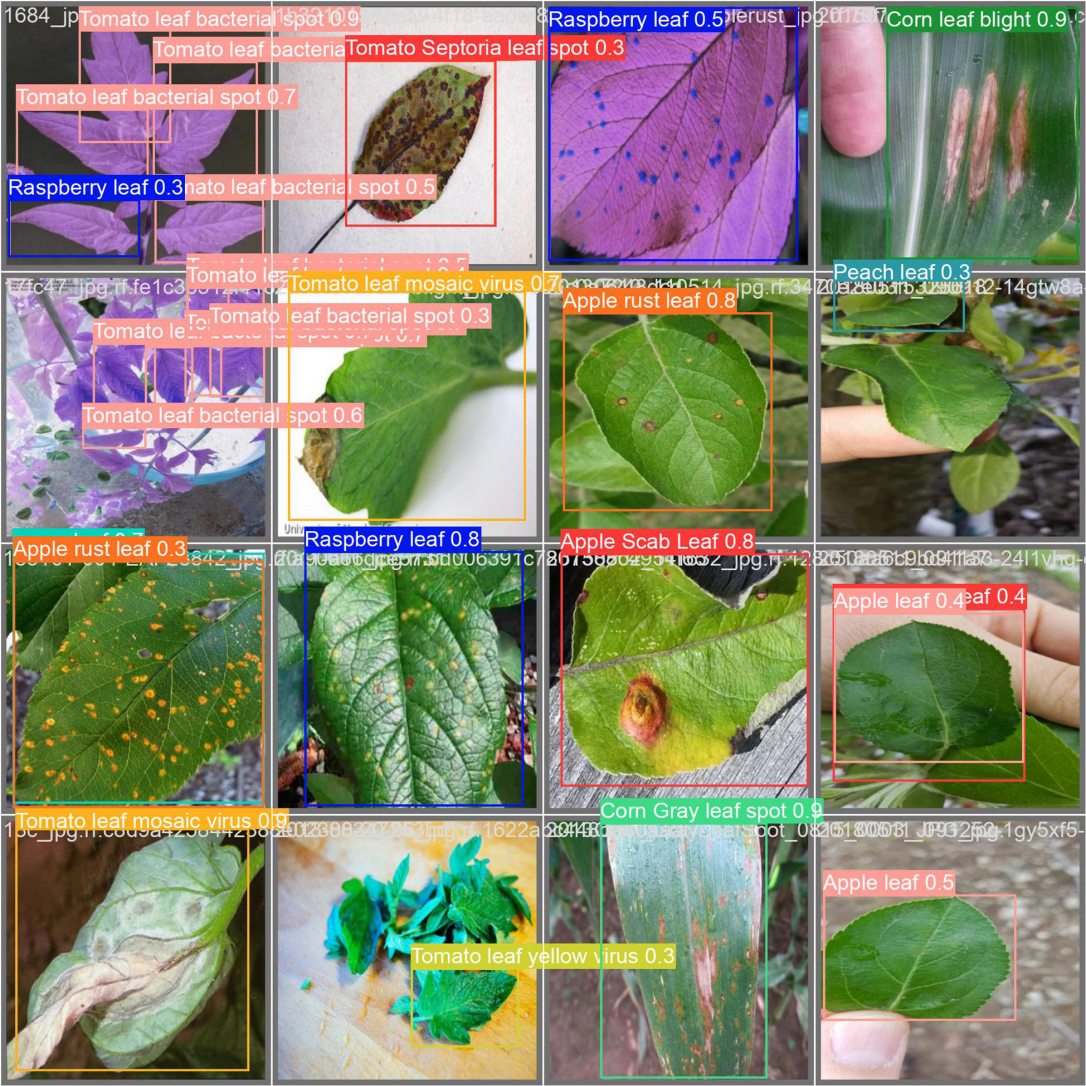
Images of both healthy plants and diseases detected using our model.

The detection samples selected in **Figure 9** are all from the test set. Across various environments, the model demonstrates strong detection capabilities, and its robustness meets practical engineering needs. However, in detection tasks involving small and densely clustered plant diseases, the model inevitably encounters some missed detections and false positives. For example, due to the similarity in color and texture between certain disease spots and surrounding healthy leaves when viewed from an overhead perspective, there is an increased likelihood of false detections; if the diseased areas on the plants are too small, they might be mistaken for background and overlooked by the model.

## 4 Discussion

This study introduces the SerpensGate framework to effectively enhance the YOLOv8 model, demonstrating significant improvements in the task of plant disease detection. Firstly, compared to the traditional YOLOv8 model, SerpensGate-YOLOv8 shows notable advancements across multiple performance metrics. Our model achieved a 3.3% increase in mean Average Precision (mAP@0.5), reaching 64.9%. This improvement is primarily attributed to the integration of the Dynamic Snake Convolution (DySnakeConv) and Super Token Attention (STA) mechanisms. These innovative technologies greatly enhance the model’s ability to detect fine, elongated, and twisted structural details while also significantly improving its global feature capture, particularly excelling in handling plant diseases in complex agricultural environments.

Additionally, the introduction of the SPPELAN technique significantly improves the model’s multi-scale feature extraction and information aggregation capabilities. SPPELAN combines the advantages of spatial pyramid pooling for capturing multi-scale information and efficient layer aggregation for better information flow and reuse. This not only improves the model’s accuracy but also optimizes inference speed, validating the necessity of multi-scale processing in plant disease detection.

Despite these advancements, the study still faces several challenges. The imbalance in dataset categories affects the model’s generalization ability for rare diseases, resulting in suboptimal performance in certain classes. Furthermore, misclassification issues persist between morphologically similar diseases (such as tomato yellow leaf curl virus and tomato leaf spot), indicating that further optimization of feature extraction and classification algorithms is needed. Lastly, environmental interferences, such as lighting variations and leaf occlusion, continue to impact model performance. Future research should aim to enhance the model’s robustness to these factors to further improve its performance.

In summary, the SerpensGate-YOLOv8 model provides an effective technological approach for plant disease detection. While it has made significant progress in terms of performance, it is still necessary to address existing limitations by expanding the dataset and optimizing algorithms. These improvements will further enhance the model’s applicability and reliability in real-world agricultural scenarios.

## 5 Conclusions

The SerpensGate-YOLOv8 model proposed in this study significantly improves detection accuracy and efficiency in plant disease detection tasks through the integration of Dynamic Snake Convolution (DySnakeConv), SPPELAN technology, and the Super Token Attention (STA) mechanism. This model not only overcomes the limitations of traditional convolutional neural networks in handling complex shapes but also optimizes the feature extraction and aggregation processes, resulting in a significant enhancement in overall performance. It achieves a mAP value of 0.649 without significantly increasing computational costs.

Compared to traditional models, SerpensGate-YOLOv8 demonstrates excellent robustness and applicability in complex agricultural environments, offering strong technical support for smart agriculture. Despite the model’s adaptability, real-world challenges such as occlusion, background blurring, and changes in lighting conditions remain. Therefore, future research will focus on expanding the dataset size, improving data annotation techniques, and further optimizing the algorithm to improve the model’s generalization capability and real-world performance.

Future work will concentrate on reducing the model’s size to improve its feasibility for real-world deployment, while continuing to optimize detection accuracy and facilitating the broader application of plant disease detection in smart agriculture.

## Data Availability

The datasets presented in this study can be found in online repositories. The names of the repository/repositories and accession number(s) can be found below: https://paperswithcode.com/dataset/plantdoc.
